# Medical Education

**Published:** 1958-04

**Authors:** A. V. Neale


### *Correspondence*

Dear Sir,

*Medical Education*

It has been said that our greatest responsibilities lie with the next and subsequent generations: there is no exception in the case of the medical profession. It is inherent in our tradition that, even though we may not transmute, we shall transmit our knowledge and experience in a Hippocratic reverence: "Pure and holy will I keep my Life and my art".

Medical Education has never been static. To thoughtful people there is nearly always some focus in need of "fundamental reconsideration" in relation to the "aim and purpose" of the medical curriculum. It is implied in Hippocrates Aphorism 1. "The art is long; the occasions sudden; the judgements difficult: neither is it sufficient (only) that the physician do his office—the patient and his attendants must do their duty and the externals must likewise be well ordered."

Studentship and practice shall, therefore, have a wholeness and a comprehension much of which is, perhaps, more nearly related to a liberal understanding and application of the humanities than to a varying (and expansive) pharmacopoeia (official and unofficial). There is nothing new in this: the only difficulty is to achieve this in the face of conflicting demands. From the earliest days we should do all we can to encourage ourselves as teachers to the discovery of an increasing unity in the apparent diversity of Nature. Medical education could more wisely be centred on a concept of human biology, and man's place in nature, and his mental health. But these general principles must be made throughly apparent to the student in his earliest acquaintance with his teachers. He can, by an infectious enthusiasm, soon come to understand that the most searching clinical problems require the greatest reference to basic science. The dichotomy—pre-clinical and clinical—is obsolete: there must be a two-way system in thought and practice. Our awareness of the dividing lines between the normal, the near-normal and the abnormal is tuned up by the marginal facts of physiology and pathology.

In my experience, the student is more attracted in clinical discussion by rather frequent and relevant reference to the normal. I sense a growing concern of the students for a much wider perspective in the patient's problems and a willingness for more searching enquiry into the basic causes of disorder. There is even an increasing interest in health, and a suggestion, here and there, that the undergraduate medical curriculum need not be a stereotyped performance with a main utilitarian aim. Education is not synonymous with training: I hope it is ever true that our students might obtain education; we can safely reserve the term training for greyhounds. The suggestion that education in the Medical Faculty may have moved somewhat towards a technological training is something which should pull us all up with some quick thinking. The idea is abhorrent and would certainly have alarmed Sir Thomas Browne who, in 1643, regarded "human material" with reverence:

> "Thus we are men and we know not how; there is something in us that can be without us and will be after us, though it is strange that it hath no history what it was before us, nor can tell how it entered us . . . it is better to sit down in a modest ignorance, and rest contented with the natural blessing of our own reasons than buy the uncertain knowledge of this life, with sweat and vexation".

One of the greatest values of University life is the freedom of access of minds: but even in the days of the student-apprentice the same stood. Thomas Sydenham (c.1675) believed "the designs to attain to the right understanding of any Art or Profession included the choosing of some Eminent Men of the Art to be the guides and to provide Pattern and by whose Direction and Example, joyned with a tolerable Capacity . . . is the best and readiest way". It was not entirely incompatible with good progress that:

> In the days of Abernethy and Cheselden and Pott
> The dutiful apprentice had a very trying lot;
> He rose at five, and washed and dried the bottles on the shelf,
> He rolled the pills and bandages and then he washed himself,
> And after years of toil and sweat and a minimum of "brass"
> One famous day he was allowed to hold a cupping glass.

Good medical education should include a spirited interest in education in health or, more formally stated, "Health Education". Faustus in his more philosophic moments considered "the end of physic is our body's health"—but in a contrary phase of boastful revelry he overstated the curative aim:

> Couldst thou make men to live eternally,
> Or, being dead, raise them to life again
> Then this profession were to be esteem'd!

Our students should come to recognise that the public can have no greater asset than a close and continued relationship with a wise, sound, general medical adviser.

It is probably fortunate that most systems of education are constantly under the fire of general criticism: medical education, however, is unlikely to suffer from much stagnation, for, whenever the lay public stops criticising the type of modern doctor, the medical profession itself may be counted on to stir up the stagnant pool and cleanse it of its sedimentary deposit. It is hoped that the suggestion that students have been taught a great deal about the mechanism of disease but very little about the practice of medicine—that they are too much "scientific" and do not know how to take care of patients and people—will be firmly refuted. The vital importance of the personal relationship between physician and patient in the practice of medicine must form the background to most other considerations: the student should be encouraged in the thought that the profession should allow of intellectual freedom, give character as much chance as cleverness and yet be subject to the "tonic of difficulty and the spice of danger".

Teachers vary—some can think but have neither tongue nor technique; some are phonographic; some have technique but neither talk nor think; and some can think, talk and work. Be that as it may, the fact remains that some of the best instructors for students may have little conception of the highest lines of work, but have an immaculate ability to convey meaningful generalities. The greatest intellectual excitement comes from teachers with first-class minds who are talking about the fundamentals of the subject. Sir William Osler considered that a man may be a good student who reads only the book of nature.

Medical Education was called by a cynic "that most boring subject", but we, as teachers, violently reject the suggestion. In fact, after reading the "Foreword" by the Vice-Chancellor and "Some Thoughts of Medical Education" in this Journal (January, 1958), we have every reason to look forward to a thorough re-awakening of the joys and betterment of the medical student's task: to hope that there may be more time for self selection of studies and that the natural rule of elective attendance will be re-established (as was the common practice throughout the whole of the "final" year 25 years ago). The students can thus, so to speak, become "free swimmers". Each student will find his own way around and about the numerous facilities available to him. In fact, by such private enterprise, he will certainly discover a real University education: something which will lead on to a satisfying maturity in his pre-registration year and ultimately to that ever-widening conception of what a doctor should be—a teacher himself, aware of humanity in its widest senses; knowledgeable that Medicine is but a part of Nature's plan, that wide fields of enquiry into the aspects of Mental, Social, and Environmental health are awaited, and last, but not least, that success often turns on the snapping up of unconsidered trifles, even in the clinical world.

Medical Education will, we hope, fall into the general ideas (as expressed by the Royal College of Physicians) of a compromise between education in its true sense—the development of habits of learning and thought and an understanding of principles—on the one hand, and the acquisition of necessary technical knowledge on the other.

Yours sincerely,

A. V. Neale.

